# Generation of human monoclonal antibodies recognising membranous antigens of the lung adenocarcinoma cell line A549 using an AMeX immunohistostaining method.

**DOI:** 10.1038/bjc.1996.366

**Published:** 1996-08

**Authors:** K. Yoshinari, H. Kimura, K. Arai, I. Sugawara, K. Noda, M. Kihara, H. Misaki, Y. Yamaguchi

**Affiliations:** Diagnostics Research and Development Department, Asahi Chemical Industry Co. Ltd., Shizuoka, Japan.

## Abstract

**Images:**


British Journal of Cancer (1996) 74, 359-367
© 1996 Stockton Press All rights reserved 0007-0920/96 $12.00

### Generation of human monoclonal antibodies recognising membranous antigens of the lung adenocarcinoma cell line A549 using an AMeX immunohistostaining method

K Yoshinari[1], H Kimura[2], K Arai[1], I Sugawara[3], K Noda[4], M Kihara[5], H Misaki[1] and Y Yamaguchi[6]

[1]*Diagnostics Research and Development Department, Asahi Chemical Industry Co. Ltd., Ohito-cho, Shizuoka;* [2]*Division of Chest Diseases, Chiba Cancer Center, Nitona-cho, Chuo-ku, Chiba;* [3]*Department of Pathology, Saitama Medical University Medical Center, Kawagoe-city, Saitama;* [4]*Department of Chest Diseases, Kanagawa Cancer Center Hospital;* [5]*Department of Epidemiology, Kanagawa Cancer Center Research Institute, Nakao-cho, Asahi-ku, Yokohama;* [6]*Department of Surgery, Institute of Pulmonary Cancer Research, School of Medicine, Chiba University, Inohana, Chiba, Japan.*

**Summary** Four monoclonal antibodies (MAbs) from hybridoma obtained by *in vitro* stimulation of regional lymph node lymphocytes from lung cancer patients and electrofusion of the stimulated cells with murine or human–mouse myeloma cells were reactive to lung cancer cells in enzyme-linked immunoabsorbent assay, and to lung cancer tissue in immunohistochemical analysis using acetone–methyl benzoate–xylene (AMeX) fixed tissue and in immunofluorescence analysis. Three of the MAbs (designated ZLG40, 27D57 and 28K29) recognised cell-surface antigens of the lung adenocarcinoma cell line A549 and the remaining one (designated 29D38) recognised nuclear membrane antigens of the same cell line. The three surface-binding MAbs showed a significant complement-dependent cytotoxicity (CDC) to the A549 cells, but the membrane-binding 29D38 showed no CDC to the A549 cells. Western blotting of the extracts of the A549 or PC6 (small-cell lung cancer) cell lines by the four MAbs showed a 28K29 antigen band at $M_r$ of approximately 600 000 ($\pm$2-ME), a ZLG40 antigen band at $M_r$ 50 000 ($\pm$2-ME), and one 29D38 antigen band at $M_r$ of more than 1 000 000 ($-$2-ME) and $M_r$ between 20 000 and 80 000 (+2-ME), but no detectable band for 27D57 antigen.

**Keywords:** human monoclonal antibody; cell surface antigen; nuclear membrane antigen; acetone–methyl benzoate–xylene (AMeX); heterohybridoma

It is widely recognised that human monoclonal antibodies (MAbs) specific for tumour-associated antigens and particularly for cell surface antigens may be useful as *in vivo* diagnostic or therapeutic tools, but efforts to obtain such antibodies have been hampered by difficulties in their detection and production (James *et al.*, 1987; Hanna *et al.*, 1991). Screening for human MAbs is most commonly done by immunohistochemical staining using formalin-fixed and paraffin-embedded tissue sections of tumour specimens, but this has generally resulted in the detection of MAbs specific to cytoplasmic rather than cell surface antigens of cancer cells (Hanna *et al.*, 1991). Alternatively, frozen sections of tumour tissues have been used for detection of membrane-binding MAbs, but their preparation is generally difficult and time-consuming and their microscopic observation is often ambiguous because of difficulties in their immobilisation on glass slides.

Sato *et al.* (1986), on the other hand, have reported efficient detection of cell surface antigens of normal lymphocytes using acetone fixation followed by clearance of the acetone with methyl benzoate and xylene, largely free of the destruction of cell surface antigens that generally occurs with formalin fixation.

In the present study, we therefore investigated the use of this AMeX method in the screening of MAbs for binding to the membranes of cells from the tissue of lung cancer patients, in addition to the conventional formalin fixation and frozen tissue methods.

Human MAbs have been generated by various techniques, generally involving: (1) fusion of murine, human–mouse or human myeloma cells, originally developed by Köhler and Milstein (1975); (2) transplanting of MAb-producing human lymphocytes to severe combined immunodeficient (SCID) mice (McCune *et al.*, 1988; Duchosal *et al.*, 1992); or (3) phage display technology or genetic manipulation techniques (McCafferty *et al.*, 1990; Zebedee *et al.*, 1992; Green *et al.*, 1994; Lonberg *et al.*, 1994). Among these methods, one of the most readily implemented is the fusion of human lymphocytes with murine or human–mouse myeloma cells, but it has commonly suffered from inefficiency in the establishment of MAb-producing hybridomas and instability in their MAb production. As previously reported, we have found it possible to increase the efficiency of establishment of such hybridoma by prior *in vitro* stimulation of the human lymphocytes with mitogens or interleukins (ILs) (James *et al.*, 1987), and to obtain stable MAb production by screening of the hybridomas for production of MAbs bearing $\lambda$-chain as the L-chain subtype (Uchiyama *et al.*, 1987; Iizasa *et al.*, 1990).

Here we describe the investigation of the effectiveness of the use of the AMeX method in screening the MAbs for binding to cell surface and nuclear membrane antigens of cancer cells.

### Materials and methods

### *Agents and cell lines*

LPS (*Escherichia coli* 0127:B8) and HAT were purchased from Sigma Chemical Co. (St Louis, MO, USA), SACI (Pansorbin cells 507861) from Calbiochem (San Diego, CA, USA), recombinant ILs and stem cell factor (SCF) from Genzyme (Cambridge, MA, USA), fetal calf serum (FCS) from Cytosystems (Castle Hill, Australia, cat. no. 15-010-0500V), and bovine insulin, human transferrin and sodium selenite from Cosmo-Bio Co. (Tokyo, Japan). The murine myeloma cell line P3X63Ag8.653, the human–mouse myeloma cell line SHM-D33, the human lung adenocarcinoma cell line A549, the human pancreas carcinoma cell line PANC-1, the human colon carcinoma cell line SW1222 and

Correspondence: K Yoshinari, Asahi Chem Ind. Co. Ltd 632-1, Mifuku, Ohito-cho, Shizuoka 410-23, Japan
Received 21 September 1995; revised 14 February 1996; accepted 23 February 1996

the human lung diploid cell line WI38 were obtained from the American Type Culture Collection (Rockville, MD, USA). The human lung adenocarcinoma cell lines PC9 and PC14, the human lung squamous carcinoma cell line QG56, the human lung large-cell carcinoma cell line PC13 and the human small-cell lung cancer cell line PC-6 were obtained from Immuno-Biological Laboratories (IBL) (Gunma, Japan). The human lung adenocarcinoma cell line PC3 and the human lung squamous carcinoma cell line EBC-1 were supplied by the Japanese Cancer Research Resources Bank (Tokyo, Japan). The ovarian carcinoma cell lines RMG-1 and RTSG were kindly supplied by Dr S Nozawa of Keio University (Tokyo, Japan).

*Lymphocytes*

Regional lymph nodes from patients with primary lung cancer were obtained aseptically at surgery. A single-cell suspension (lymphocytes) in 10% FCS-supplemented RPMI-1640 (Flow Laboratories) medium was prepared by passing the lymph nodes through sterile steel mesh (no. 200) after teasing with scissors.

In vitro *stimulation of lymphocytes*

Lymphocytes ($2.5 \times 10^6$ cells $ml^{-1}$) prewashed with PBS were resuspended in RDF medium (a 2:1:1 mixture of RPMI-1640, DMEM and Ham F12) (Murakami *et al.*, 1985) with 10% FCS and 50 $\mu$M 2-mercaptoethanol (2-ME). The cell suspension was pipetted at 2 ml per well into 6-well tissue culture plates and mitogens and ILs were added to the wells as indicated in Table I, followed by incubation at 37°C in humidified 5% carbon dioxide air for the times indicated in the table.

*Cell fusion*

Electrofusion of lymphocytes and mouse or human–mouse myeloma cells was performed by the method of Foung *et al.* (1990) and Zimmermann *et al.* (1990) with some modifications. Briefly, mixed cells (lymphocytes–myeloma cells = $5 \times 10^6$: $5 \times 10^6$) were washed once with iso-osmolar fusion medium (300L3; 280 mM sorbitol, 0.1 mM $Ca^{2+}$ acetate, 0.5 mM $Mg^{2+}$ acetate and 1 mg $ml^{-1}$ BSA). After collection by low-speed centrifugation, cell pellets were resuspended in 2.5 ml of hypo-osmolar fusion medium (75L3; 70 mM sorbitol, 0.1 mM $Ca^{2+}$ acetate, 0.5 mM $Mg^{2+}$ acetate, and 1 mg $ml^{-1}$ BSA). Five minutes later, 0.83 ml aliquots of the cell suspension were placed in fusion chambers and exposed to one rectangular electrical pulse (1 MHz, 1.00 kV $cm^{-1}$, 15 $\mu$s) in the model SSH-1 cell fusion apparatus (Shimadzu Corporation, Kyoto, Japan). After another 5 min standing, the three aliquots of cell suspension (total volume 2.5 ml) were pipetted into 30 ml of HAT medium (RDF medium supplemented with 100 $\mu$M hypoxanthine, 16 $\mu$M thymidine, 0.2 $\mu$M aminopterin, 5 $\mu$g $ml^{-1}$ bovine insulin, 5 $\mu$g $ml^{-1}$ human transferrin, 5 ng $ml^{-1}$ sodium selenite, 50 $\mu$M 2-ME, 20 U $ml^{-1}$ IL-6, 40% BALB/c mouse splenocyte culture supernatant and 10% FCS) and then distributed in 0.1 ml portions into 96-well culture microplates. Plates were incubated for 2–4 weeks in humidified 5% carbon dioxide air at 37°C. Wells with growing colonies were selected microscopically, production of secreted immunoglobulin (Ig) was measured by a sandwich ELISA and Ig-secreting wells were maintained in HT medium (RDF medium supplemented with 100 $\mu$M hypoxanthine, 16 $\mu$M thymidine, 5 $\mu$g $ml^{-1}$ bovine insulin, 5 $\mu$g $ml^{-1}$ human transferrin, 5 ng $ml^{-1}$ sodium selenite, 50 $\mu$M 2-ME, 20 U $ml^{-1}$ IL-6 and 10% FCS).

*Cell culture and adaptation to serum-free medium*

Heterohybridomas secreting Ig with $\lambda$-chain were screened in immunohistological and immunofluorescence methods as described below. The selected hybridomas secreting Ig reactive with lung cancer tissues and cell membrane or nuclear membrane of A549 cells were recloned three times by limiting dilution (0.5–2 cells $ml^{-1}$) in HT medium and finally adapted to a serum-free medium, Hybridoma-SFM (Gibco-BRL) supplemented with penicillin (50 IU $ml^{-1}$) and streptomycin (50 $\mu$g $ml^{-1}$). For 28K29 and 28B49 (a negative control) cells, HT was further added to the serum-free medium.

*Measurement of Ig production and determination of Ab class*

Human MAb was measured by a sandwich ELISA, in which 96-well ELISA microplates (Sumitomo Bakelite, Tokyo, Japan) precoated overnight at 4°C with 2000-fold-diluted goat antibodies to human Ig (Tago no.4103, Tago Inc., Burlingame, CA, USA) were blocked for 1 h at room temperature with 2% BSA in PBS and washed twice with PBS. Supernatants from the cell cultures, in serial 2-fold dilutions by 0.2% BSA–PBS, were transferred to the wells and incubated for 1 h at 37°C. The wells were then washed three times, and bound MAb was detected by incubation for 30 min at 37°C with one of the following 1000-fold-diluted alkaline phosphatase-conjugated goat Ab $F(ab')_2$ anti-human chains in 0.2% BSA–PBS: IgM $\mu$ (Tago no.4602); IgG $\gamma$ (Tago no.4600); IgA $\alpha$ (Tago no.4601); Ig $\lambda$ (Tago no.4608); Ig $\kappa$ (Tago no.4606). The plates were then washed three times, followed by coloration with *p*-nitrophenyl phosphate (Sigma) in 0.1 M diethanolamine buffer (pH 9.8) and human MAb quantitation with human IgM (Cappel no.6001–1590, Organon Technica Corporation, West Chester, PA, USA), IgG (Cappel no.6001–0080) and IgA (Cappel no.6001–0020) as the standards.

*Cell ELISA*

Wells with various cells grown subconfluently in 96-well microplates (Sumitomo Bakelite Co., Tokyo, Japan) were washed once with PBS, followed by standing for 15 min at room temperature in PBS containing (0.05% glutaraldehyde for cell fixation (Dorreen *et al.*, 1982). Then, the wells were washed three times with PBS containing 0.05% Tween 20 (Tween 20/PBS) and blocked with PBS containing 3% BSA. After washing five times with PBS, 50 $\mu$l aliquots of MAb-containing samples were dispensed into each well. After incubation for 3 h at room temperature, the wells were washed three times with Tween 20/PBS, followed by the addition of alkaline phosphatase (ALP)-conjugated goat $F(ab')_2$ anti-human Ig (Tago) diluted 1:1000 with 0.2% BSA–PBS. After incubation for 1 h, the wells were washed

**Table I** Conditions of *in vitro* stimulation of human lymphocytes

| *Hybridoma* | *Ab class* | *Histology*[a] | *Myeloma* | *Mitogen* | *Lymphokines*[b] | *Incubation (days)* |
|---|---|---|---|---|---|---|
| ZLG40 | IgM, $\lambda$ | Sq | P3X63Ag8.653 | LPS (20 $\mu$g $ml^{-1}$) | IL-4, 6 | 8 |
| 27D57 | IgM, $\lambda$ | Sq | P3X63Ag8.653 | LPS (20 $\mu$g $ml^{-1}$) | IL-4 | 5 |
| 28K29 | IgM, $\lambda$ | Ad | SHM-D33 | SACI (1/10000) | None | 5 |
| 29D38 | IgM, $\lambda$ | Sq | P3X63Ag8.653 | LPS (20 $\mu$g $ml^{-1}$) | IL-1, 4, 7, SCF | 5 |
| 28B49 | IgM, $\lambda$ | Ad | P3X63Ag8.653 | LPS (20 $\mu$g $ml^{-1}$) | IL-1, 4 | 5 |

[a]Sq, squamous carcinoma; Ad, adenocarcinoma. [b]IL-4, 100 u $ml^{-1}$; IL-6, 100 u $ml^{-1}$; IL-7, 100 u $ml^{-1}$; SCF, 50 ng $ml^{-1}$.

three times and detection was accomplished using 100 μl of a 0.67 M p-nitrophenylphosphate in 1 M diethanolamine buffer (pH 9.6) containing 0.5 mM magnesium chloride. After 10 min incubation, the reaction was stopped with 100 μl of 1 N sodium hydroxide and the reactivity of each MAb to the cells was determined by measuring the absorbance at 405 nm.

*Immunohistochemical staining*

Sections (5 μm) of lung tissues taken at surgery from lung cancer patients were prepared by three different methods and stained as follows.

In the preparation of frozen sections, tissues were placed in Cryomold (Miles, Elkhart, IN, USA), covered with optical cutting temperature (OCT) compound (Miles Inc.) and immediately frozen in iso-pentane over liquid nitrogen and stored at −80°C. Just before staining, cryostat sections (5 μm) were taken and placed onto precleared, 0.02% poly-L-lysine ($M_r$ < 150 000, Sigma)-treated microscope slides, where they were fixed in 0.1 M phosphate buffer containing 4% paraformaldehyde (PFA) for 20 min at room temperature and then washed three times in PBS.

To prepare AMeX-fixed sections (Sato *et al.*, 1986), tissues were first fixed in acetone precooled to 4°C and stored at −20°C for 1–3 days. On removal from storage they were dehydrated by two cycles of acetone, followed by acetone removal in two cycles of 15 min immersion in methyl benzoate followed by 15 min in xylene, and then embedded in paraffin by the usual procedure. Sections (5 μm) of the paraffin-embedded tissues were taken, deparaffinised by xylene and acetone, and fixed in 4% PFA for 5 min. They were then prepared for staining by washing in water and PBS.

Formalin-fixed sections were prepared in the usual manner. Tissues were fixed in 10% formalin solution and embedded in paraffin, and sections (5 μm) of the embedded tissues were taken and then deparaffinised by xylene and ethanol in series and then washed in water and PBS.

In all three cases, immunostaining was performed as follows. The washed tissue sections were blocked with 10% normal goat serum (Nichirei, Tokyo, Japan) for 20 min at room temperature, followed by the addition of MAb (0.5–20 μg ml$^{-1}$) test samples. After incubation for 18 h at 4°C in a moisturised atmosphere, the sections were washed three times with PBS. Peroxidase-conjugated goat F(ab′)$_2$ anti-human Ig (Cappel no. 3301–0231) diluted 1:1000 (v/v) in 0.2% BSA–PBS was layered on the washed sections. After incubation for 2 h at room temperature and washing three times with PBS, the sections were colorated with a substrate solution for peroxidase (0.02% diaminobenzidine and 0.005% hydrogen peroxide in 50 mM Tris-HCL buffer, pH 7.6), incubated for 5 min, washed with water, and counterstained with Harris-type haematoxylin solution (Sigma). The sections were then washed thoroughly with tap water and dehydrated with a graded series of ethanol and xylene. A cover glass was mounted on each slide with xylene-based Mount Quick (Daido-Sangyou, Tokyo, Japan), for microscopical observation.

*Immunofluorescence*

A549 cells grown subconfluently in DMEM containing 10% FCS in T-75 culture bottles were incubated with PBS containing 0.02% EDTA for 10–20 min, then scraped and collected by centrifugation at low speed. Aliquots of 2 × 10$^5$ A549 cells were resuspended in 150 μl supernatant containing MAb (0.5–20 μg ml$^{-1}$), followed by incubation for 2 h at 4°C. The cells were pelleted by centrifugation at low speed and resuspended in 100 μl of FITC-conjugated goat F(ab′)$_2$ anti-human Ig (Cappel). After incubation for 1 h at 4°C, the cells were washed three times with PBS and resuspended in 50 μl of PBS for observation with an Axioscop (Zeiss, Germany) fluorescence microscope.

*CDC activity*

A549 cells grown subconfluently in 10% FCS-supplemented DMEM in four T-75 culture bottles were collected as described above for immunofluorescence, and resuspended at a density of 1 × 10$^6$ cells ml$^{-1}$ in DMEM devoid of phenol red in the absence of FCS. Aliquots (100 μl) of the cell suspension were dispensed in 96-well microplates and 10 μg ml$^{-1}$ of each MAb and 10% low-toxic rabbit complement (Cederlane, Ontario, Canada) were added to each well, followed by incubation for 2 h at 37°C in humidified 5% carbon dioxide air. Viable cells were then counted by colorimetric assay using sodium 3′-[1-(phenylamino-carbonyl)-3,4-tetrazolium]-bis (4-methoxy-6-nitro) benzene sulphonic acid (XTT), Cell Proliferation kit II (Boehringer Mannheim, Germany) (Stevens *et al.*, 1993; Buttke *et al.*, 1993). A 50 μl solution of XTT was next added to each well and incubation was continued for another 4 h. Absorbance at 450 nm was then measured, and CDC activity was expressed as the percentage difference between incubations with and without MAb.

*Preparation of cell extracts*

Subconfluent A549 or PC-6 cells grown in 5% FCS-supplemented RDF medium in four T-75 culture bottles were collected as described in the section on immunofluorescence. Approximately 5–10 × 10$^7$ cells were resuspended in 1 ml of PBS containing 1% Triton X100, 5 μg ml$^{-1}$ leupeptin, 5 μg ml$^{-1}$ chymostatin, 5 μg ml$^{-1}$ pepstatin and 1 mM phenylmethylsulphonyl fluoride (PMSF), and incubated for 45 min on ice with intermittent shaking. Supernatants were collected by centrifugation at 27 000 *g* for 5 min, detergent was removed with an SM-2 Econo-column (Japan Bio-Rad, Tokyo, Japan), and the cell extracts were used for immunoblotting.

*Biotin labelling of MAb*

MAb solution purified from supernatants (2 l) of hybridoma grown in a serum-free medium using a hydroxylapatite column was buffer-exchanged to 50 mM bicarbonate buffer (pH 8.5) and concentrated to 2 mg ml$^{-1}$ with a Centricon-30 (Millipore). An aliquot (74 μl) of 1 mg ml$^{-1}$ NHS-LC-biotin (Pierce, Rockford, IL, USA) was added, followed by incubation for 2 h on ice. The reaction mixture was centrifuged (10 000 *g*, 30 min) with a Centricon-30 to remove free biotin, and the concentrated solution was diluted in 10 mM phosphate buffer containing 150 mM sodium chloride and 0.1% sodium azide. The incubation–

**Figure 1** Reactivity of MAbs to glutaraldehyde-fixed A549 cells. All MAbs except the 28B49 isotype-matched negative control reacted with the glutaraldehyde-fixed A549 cells (a lung adenocarcinoma cell line). Neither 28B49 MAb nor the other negative control, hIgM (polyclonal Ab, Cappel), reacted with the cells. All data shown as means ± s.d. (bar) of three data points.

centrifugation–dilution cycle was performed three times, to obtain biotinylated MAb for use in immunoblotting.

*Immunoblotting*

Detergent-free extracts of A549 or PC-6 cells were diluted 5-fold with an SDS sample buffer [0.0625 M Tris-HCl buffer (pH 6.8) containing 10% glycerol, 2% SDS and 0.005% bromophenol blue with or without 5% 2-ME] and boiled for 3 min. After rapid cooling, 5 μl was applied on each lane of 2–15% or 4–20% SDS-polyacrylamide gel and electrophoresed by the method of Laemmli (1970). Electroblotting from the gel to Immobilon-P membrane (Millipore) was performed (Towbin *et al.*, 1979) using an SDS-PAGE buffer containing 20% methanol on a semi-dry blotting apparatus (Bio-Rad). After electroblotting, the membranes were blocked with 0.02% Tween 20/PBS containing 0.5% skim milk for 30 min at room temperature. Biotinylated MAb (1 μg $ml^{-1}$) diluted in 0.02% Tween 20/PBS containing 0.5% skim milk was added and incubated for 1 h, followed by washing three times with 0.02% Tween 20/PBS. An alkaline phosphatase-conjugated streptavidin (Jackson ImmunoResearch Laboratories) solution diluted 1:500 (v/v) in 0.02% Tween 20/PBS containing 0.05% skim milk was added, and incubation was continued for 30 min. The membranes were washed three times with 0.02% Tween 20/PBS, followed by coloration using 10 ml of 0.5 M diethanolamine (pH 9.8) supplemented with 30 μl each of BCIP (50 mg $ml^{-1}$ of dimethylformamide) and NTB (75 mg $ml^{-1}$ of 70% dimethylformamide). The reaction was stopped by washing with water.

*N-terminal amino acid sequencing*

Aliquots (20 μg) of each MAb were electrophoresed on a 12.5% isocratic SDS-polyacrylamide gel under reducing conditions, and proteins on the gel were blotted to Immobilon[PSQ] (Millipore) PVDF membrane by the method

**Figure 2** Immunofluorescence staining patterns in the lung adenocarcinoma cell line A549. Indirect immunofluorescence staining of A549 cells shows positive reactivity of MAbs to the cell surface (**a**, 28K29 MAb; **b**, ZLG40 MAb; **c**, 27D57 MAb) and to the nuclear membrane (**d**, 29D38 MAb). The negative control 28B49 MAb did not react with the cells (**e**). (× 300).

Table II Cell ELISA using various human cell lines

| Cell line (histology) | ZLG40 | 27D57 | 28K29 | 29D38 | 28B49 |
|---|---|---|---|---|---|
| PC3 (lung adenocarcinoma) | – | – | + | – | – |
| PC9 (lung adenocarcinoma) | + | – | + + | ± | – |
| PC14 (lung adenocarcinoma) | + | + | + + | ± | – |
| A549 (lung adenocarcinoma) | + | + | + + | + | – |
| EBC-1 (lung squamous carcinoma) | + | – | + + | – | – |
| QG56 (lung squamous carcinoma) | + | + | + + | ± | – |
| PC13 (large-cell lung carcinoma) | + | + | + + | + | – |
| PC6 (small-cell lung carcinoma) | + + | + | + | + + | – |
| PANC-1 (pancreas carcinoma) | + | + + | + | – | – |
| SW1222 (colon carcinoma) | + | ± | + | – | – |
| RMG-1 (ovarian carcinoma) | + | + + | + | – | – |
| RTSG (ovarian carcinoma) | + | + + | + | – | – |
| WI38 (lung diploid fibroblast) | + | ± | + | – | – |

Cell ELISA using various cells was performed as described in Materials and methods. MAb reactivity with each cell line was classified into negative (–), and weakly (±), moderately (+) and strongly (+ +) positive on an arbitrary scale.

of Hirano et al. (1990). After staining of the blotted membrane with Coomassie brilliant blue solution, heavy and light chains of each MAb were excised for analysis of N-terminal amino acid sequences with the Protein Sequencer[PSQ] (Shimadzu Corporation, Kyoto, Japan).

### Results

### Preliminary generation and screening

Electrofusion of the regional lymph node lymphocytes from seven patients with primary lung cancer, stimulated with mitogens or mitogens plus ILs, and the murine myeloma cell line (P3X63Ag8.653) or the human–mouse heteromyeloma cell line (SHM-D33) resulted in the generation of 2114 Ig-producing hybridomas. The MAbs produced by these hybridomas were screened for (1) constituent λ-chain; (2) reactivity in cell ELISA to the glutaraldehyde-fixed A549 lung adenocarcinoma cell line; (3) immunofluorescent staining of the A549 cell or nucleus membrane; and (4) immunohistochemical staining of the cancerous but not the non-cancerous parts of lung cancer tissue sections prepared by freezing, formalin fixation or AMeX fixation. Nine of the Ig-producing hybridomas satisfied all of these criteria, and four were prepared for the present study by recloning them three times and adapting them to the serum-free medium Hybridoma-SFM (GIBCO).

Table I shows the lymphocytes, mitogens and ILs used to obtain these four hybridomas secreting human MAbs (ZLG40, 27D57, 28K29 and 29D38) showing specificity to lung cancer cells and tissue, together with those used to obtain the hybridoma cell line 28B49 as an isotype-matched negative control. As shown in Figure 1, the four MAbs (1.0 $\mu g\ ml^{-1}$) selected from the screening all reacted with glutaraldehyde-fixed A549 cells grown in 96-well microplates, while the two controls 28B49 (IgM, λ) and hIgM (human polyclonal IgM) showed no reactivity to the cells. Table II shows the results of cell ELISA using various human cell lines.

As illustrated in Figure 2a–d, immunofluorescent staining showed three of the four selected MAbs (28K29, 27D57 and ZLG40) to recognise A549 cell surface antigen, and the fourth MAb (29D38) recognised nuclear membrane antigen of the same cell line. No cellular fluorescence was observed with the control MAb, 28B49 (Figure 2e).

### Immunohistochemical study

The results of immunohistochemical staining with AMeX, frozen and formalin fixation are summarised in Table III. The MAb 28K29 reacted with most of the tissue sections of lung cancer tested, regardless of the fixation method, but the other three MAbs (ZLG40, 27D57, 29D38) reacted with tissue sections fixed by freezing and by AMeX, but not with those fixed by formalin.

Representative patterns of the staining with AMeX are shown in Figure 3. All four MAbs reacted with the tissue sections fixed by this method. AMeX fixation resulted in a clear staining of cytoplasmic rather than cell surface regions (Figure 3a,c,d and e) by all four MAbs, and also an apparent staining of the luminal regions of membranes by the MAb 28K29 (Figure 3b), indicating that the corresponding antigens to the four MAbs are localised in both membranous and cytoplasmic regions of cancer cells.

The four MAbs were also tested for immunoreactivity in frozen sections of normal mammary gland, stomach, lung and kidney tissues, with negative results in all cases. None of the four stained any of the cells of normal mammary ductal, gastric epithelial, bronchiolar epithelial or proximal tubular tissues (data not shown).

Table III Immunohistostaining of lung cancer tissue sections

| Antibody | Frozen Ad | Frozen Sq | AMeX Ad | AMeX Sq | Formalin Ad | Formalin Sq |
|---|---|---|---|---|---|---|
| 28K29 | 2/3 | 2/2 | 3/3 | 2/3 | 3/3 | 3/3 |
| 27D57 | 2/3 | 2/2 | 2/3 | 1/3 | 0/3 | 0/3 |
| ZLG40 | 2/3 | 1/2 | 1/3 | 3/3 | 0/3 | 0/3 |
| 29D38 | 2/3 | 2/2 | 3/3 | 3/3 | 0/3 | 0/3 |
| 28B49 | 0/3 | 0/2 | 0/3 | 0/3 | 0/3 | 0/3 |
| hIgM | 0/3 | 0/2 | 0/3 | 0/3 | 0/3 | 0/3 |

Lung adenocarcinoma and lung squamous carcinoma tissue were fixated by the freezing, AMeX and formalin methods; number of sections with positive staining/total number of sections tested.

Table IV N-terminal amino acid sequences

| | |
|---|---|
| H chain | |
| 28K29 | N-blocked |
| 29D38 | N-blocked |
| 27D57 | EVQLV-QSGAQ-VKKPG-EQLKI- |
| ZLG40 | EVQLV-QSGAQ-VXPPG- |
| 28B49 | EVQLV-QSGGV-LVQPG- |
| V(H) | -EVQLV-QSGAE-VKKPG-ESLKI- |
| L Chain | |
| 28K29 | N-blocked |
| 29D38 | SYELT-QPPSV-SVSPG-QTARI-TXEGD-ALPK |
| 27D57 | SYELT-QPPSV-SVS |
| ZLG40 | SYELT-QP |
| 28B49 | N-blocked |
| Ig (λ) | -SVELT-QPPSG-SVSPG-KTARI-TCSGD-ALPKK- |

N-terminal amino acid sequences of five MAbs including the negative control 28B49 were determined. The known sequences of human germline V(H) (Berman et al., 1991) and human Ig (λ) (Combriato et al., 1991) with high homology to the MAbs sequenced in this study were comparatively aligned.

**Figure 3** Immunoperoxidase staining patterns in AMeX-fixed tissue sections of lung adenocarcinoma. AMeX-fixed and paraffin-embedded sections (5 $\mu$m thick) of lung adenocarcinoma tissues (ALA1 and ALA2) were reacted for reactivity of MAbs. 28K29 MAb shows positive reactivity to the cytoplasm parts of tissue section (**a,** ALA1 tissue section) and to the luminal membranes of another tissue section (**b,** ALA2). Similarly, the cytoplasmic parts of ALA1 tissue section (**a**) and to the luminal membranes as well as the cytoplasm of ALA2 (**b**). ZLG40 MAb (**c**), 27D57 MAb (**d**) and 29D38 (**e**) reacted with the cytoplasm of ALA1 tissue sections. No positive staining of ALA1 section was observed in case of 28B49 (**f**) ($\times$170).

*CDC activity*

The complement-dependent cytotoxicity of the four MAbs, as determined by incubating the A549 adenocarcinoma cells with the MAb (1 $\mu$g ml$^{-1}$) and 10% rabbit complement for 2 h at 37°C, counting the residual viable cells by the XTT method (Stevens *et al.*, 1993; Buttke *et al.*, 1993), and calculating the CDC on this basis, is shown in Figure 4. The CDC was high with the three cellular membrane-binding MAbs (approximately 30% with 28K29 and ZLG40 and approximately 12% with 27D57), but was only 3–6% with the nuclear membrane-binding MAb 29D38 and with the negative control MAb 28B49, and thus practically indistinguishable from background level CDC.

*Antigen immunoblotting*

Antigens for three of the four MAbs were identified by immunoblotting, as shown in Figure 5. The cell extracts were obtained from the lung adenocarcinoma cell line A549 and from the small-cell lung carcinoma cell line PC-6 by exposure to the detergent 1% Triton X100 and its removal with an SM-2 column. The cell extracts were run on 2–15% or 4–20% SDS-polyacrylamide gel, and the electrophoresed gel was blotted to PVDF membrane for the detection of bands immunoreactive to each MAb. The MAbs 28K29 and ZLG40 reacted with bands of approximately 600 000 and 50 000, respectively, under both non-reducing and reducing conditions. The MAb 29D38 bound to one or more substances of more than 1 000 000 at the gel top under non-reducing conditions, and to several bands between 20 000 and 80 000 under reducing conditions. The MAb 27D57 did not react with any band either the A549 or the PC-6 cell extract (data not shown).

*N-terminal amino acid sequences*

Table IV shows the results of the N-terminal sequencing of the four cell-reactive MAbs and the negative control 28B49, as determined by SDS-PAGE and the blotting to PVDF membrane followed by analysis with an automatic amino acid sequencer. The N-terminal amino acids of both the H-chain and the L-chain of MAb 28K29 were found to be blocked. The N-terminal amino acid sequences of the L-chains of 27D57, ZLG40 and 29D38 were identical as far as examined. As also shown in Table IV, a search of known amino acid sequences showed a high homology between the N-terminal amino acid sequences of the H-chains of MAbs 27D57 and ZLG40 and the human germline V (H) (Berman *et al.*, 1991), and between those of the L-chains of MAbs 29D38, 27D57 and ZLG40 and human Ig ($\lambda$) (Combriato *et al.*, 1991).

**Figure 4** CDC activity of MAbs (1 $\mu$g ml$^{-1}$) against adenocarcinoma A549 cells in the presence of 10% rabbit complement. A549 cell surface-binding MAbs (28K29, 27D57, and ZLG40) show significant CDC activity. A549 nuclear membrane-binding MAb (29D38) and two negative control Abs (28B49 MAb and hIgM) do not show CDC activity. All data shown as mean±s.d. (bar) of three data points.

### Discussion

The primary objective of the present study was the establishment of heterohybridoma cell lines producing human MAbs specific for cell-surface or nuclear membrane antigens of the lung adenocarcinoma cell line A549, from hybridomas generated by electrofusing the lymph node lymphocytes of lung cancer patients, stimulated *in vitro* with mitogens or mitogens plus lymphokines, with the readily available murine or human–mouse myeloma cells. A secondary objective, which proved important to the successful screening for such hybridomas, was the application of the AMeX method of Sato *et al.* (1986) to the fixation of solid tumour specimens consisting of cancerous tissues from lung cancer patients, to permit efficient detection of MAbs bound to cell-surface or nuclear membrane antigens.

In the effort to obtain human MAbs specific for tumour-associated antigens, immunohistochemical methods are generally preferable for the detection of cell-surface-binding MAbs. Among the several methods of tumour tissue fixation available for this purpose, formalin fixation has been most commonly employed because formalin-fixed tissue sections are easy to handle and familiar to many pathologists, even though it tends to involve inactivation or aggregation of the target antigens. Freezing has been used as an alternative method to avoid these problems, but immunohistochemical investigation of frozen tissue sections is often hindered by their fragility under the immunohistochemical staining procedure and by their incomplete or insecure immobilisation for microscopic observation.

The AMeX method was originally developed for the fixation of cell surface antigens of lymphocytes, but its adoption in the present study for the fixation of tissue sections of solid lung cancers showed it to be highly effective in screening for MAbs that recognise cell-surface or nuclear membrane antigens, as three of the four MAbs later

**Figure 5** Immunoblotting analysis of antigens. Cell extracts of PC6 (lanes 1 and 2) and A549 (lanes 3–6) were run on SDS-PAGE followed by Western blotting (lanes 1 and 2, 2–15% acrylamide gel; lanes 3–6, 4–20% acrylamide gel). The MAbs used were 28K29 (lane 1 and 2), ZLG40 (lane 3 and 4) and 29D38 (lanes 5 and 6). Lanes 1, 3 and 5 were obtained under non-reducing conditions and lanes 2, 4 and 6 under reducing conditions.

confirmed to be reactive with the A549 cells showed positive reactivity to tissue prepared by AMeX fixation (and also to those prepared by freezing), but not to tissue prepared by formalin fixation.

The present study also served to confirm the usefulness of LPS for stimulation of human lymphocytes before electrofusion to obtain hybridoma for MAb production. Many investigators have reported on the *in vitro* stimulation of human lymphocytes for this purpose (James *et al.*, 1987), but generally by SACI rather than LPS, as it is known that B-cells are activated by the polyclonal human B-cell activator SACI (Ruuskanen *et al.*, 1980; Schuurman *et al.*, 1980) but not by the polyclonal murine B cell activator LPS (Umetsu and Geha, 1987).

In our own studies, however, we have chosen to employ LPS as well as SACI because regional lymph node lymphocytes, as our source of B cells, also include immune cells such as T cells and monocytes, and it therefore seemed reasonable to presume that indirect stimulation of human B cells might occur in the presence of LPS, probably via activation of monocytes which have been shown to have the cell-surface marker CD14, which is known as a receptor for LPS (Ziegler-Heitbrock *et al.*, 1993). The results of the present study indicate that LPS is effective for such activation, at least when used in combination with lymphokines such as IL-4, IL-6 and IL-7. On this basis, we have now begun a systematic study to determine the combinations of mitogens and lymphokines most effective for this purpose (Yoshinari *et al.*, 1996).

The productivity of the four hybridomas secreting reactive MAbs, following their recloning three times and adaptation to the serum-free medium Hybridoma-SFM (GIBCO-BRL), was: 29D38, 20–40 $\mu g\ ml^{-1}$; 28K29 and 27D57, 5–20 $\mu g\ ml^{-1}$; and ZLG40, 0.5–2 $\mu g\ ml^{-1}$. Their MAb production in cell culture was found to be stable in the long term, for periods of up to 18 months or more. It may be noted, also, that the same hybridomas were found to be capable of producing MAbs in the ascites fluid of SCID mice following i.p. injection, at production levels of: 29D38, 20–40 mg per mouse; 28K29 and 27D57, 1–3 mg per mouse; ZLG40, less than 1 mg per mouse.

The results of the immunoblotting analyses provide certain insights into the identity and nature of the antigens for three of the four reactive MAbs. The analyses showed the 28K29 antigen to have $M_r$ of approximately 600 000 on SDS-PAGE under both non-reducing and reducing conditions, thus suggesting that it may be a mucin-type tumour-associated antigen (Rughetti *et al.*, 1993; Tytgat *et al.*, 1994). The ZLG40 antigen had $M_r$ of 50 000 under both non-reducing and reducing conditions. Cytokeratins of 40–68 000 (Moll *et al.*, 1982; Ramaekers *et al.*, 1983; Haspel *et al.*, 1985; Broers *et al.*, 1988; Pomato *et al.*, 1989; Erb *et al.*, 1992) have been proposed as possible tumour-associated antigens, among which cytokeratins nos. 14 and 15 have $M_r$ of 50 000. Against this, however, it must be noted that the immunofluorescence analyses showed the ZLG40 antigen to be localised at the cell surface of the A549 cells, thus suggesting that the ZLG40 antigen is not a cytokeratin as mentioned above.

The 29D38 antigen had $M_r$ of more than 1 000 000 under non-reducing conditions and between 20 000 and 80 000 in several major immunoreactive bands under reducing conditions. Recent findings show that 26S proteasome localised in the cytoplasm of cells has $M_r$ of 2 000 000 and is comprised of several protein components ranging from 22 000 to 110 000 $M_r$ (Peters *et al.*, 1993, 1994; Rechsteiner *et al.*, 1993; Rivett, 1993; Kristensen *et al.*, 1994). The immunofluorescence analysis, however, again showed that the 29D38 antigen was localised at the nuclear membrane of A549 cells, and thus suggested that the antigen would not be a proteasome itself, but that it may be a proteasome-like substance which is closely associated to tumour antigens. On the other hand, 27D57 antigen was not detected in cell extracts of A549 and PC6 cells. In summary, the results of the present study provide a basis for further study and characterisation of the antigens to three of the four MAbs, the exception being 27D57 antigen, which was not detected in the cell extracts of either A549 or PC6 by immunoblotting.

From the biological point of view, the results of the CDC activity were consistent with those of the immunofluorescence analyses. The three MAbs that bound to the cell surface of the A549 cells showed significant CDC to A549 cells, in varying degrees, while the MAb that bound to the nuclear membrane showed no CDC to A549 cells. In a related study now in progress, moreover, preliminary data on the biodistribution of the same human MAbs injected i.v. into A549 tumour cell burden SCID mice show significant accumulation of all four MAbs in the proliferated sites of A549 cells following their s.c. injection, but no such accumulation of a negative control MAb.

In conclusion, four human MAbs specific for cell surface or nuclear membrane of the lung adenocarcinoma cell line A549 were found and the heterohybridomas generating these MAbs were established, following their selection in an extensive screening of MAbs for $\lambda$-chain constituents of their L-chain and reactivity to tissue sections of lung cancer prepared by AMeX fixation. The four heterohybridomas thus obtained were shown to be capable of efficient, stable long-term production of these MAbs, and AMeX fixation was shown to facilitate the detection of MAbs specific for cell surface and nuclear membrane antigens whose detection has heretofore been difficult or impossible.

### Abbreviations

Ab, antibody; MAb, monoclonal antibody; LPS, lipopolysaccharide; SACI, *Staphylococcus aureus Cowan I*; IL, interleukin; DMEM, Dulbecco's minimum essential medium; FCS, fetal calf serum; BSA, bovine serum albumin; PFA, paraformaldehyde; SCLC, small-cell lung carcinoma; Ad, adenocarcinoma; Sq, squamous carcinoma; AMeX, acetone–methyl benzoate–xylene; 2-ME, 2-mercaptoethanol; CDC, complement-dependent cytotoxicity; SDS-PAGE, sodium dodecyl sulphate-polyacrylamide gel electrophoresis; XTT, sodium 3′-[1-(phenylamino-carbonyl)-3,4-tetrazolium]-bis(4-methoxy-6-nitro) benzene sulphonic acid.

### Acknowledgements

We thank Shigeru Ikuta and Kunio Matsumoto for helpful discussion, Tomomi Akiyama-Usami and Iyo Kawai for technical assistance, and OM Stever for critical reading of the manuscript.

### References

BERMAN JE, HUMPHRIES CG, BARTH J, ALT FW AND TUCKER PW. (1991). Structure and expression of human germline $V_H$ transcripts. *J. Exp. Med.*, **173**, 1529–1535.

BROERS JLV, RAMAEKERS FCS, ROT MK, OOSTENDORP T, HUYSMANS A, VAN MUIJEN GNP, WAGENAAR SSC. AND VOOIJS GP. (1988). Cytokeratins in different types of human lung cancer as monitored by chain-specific monoclonal antibodies. *Cancer Res.*, **48**, 3221–3229.

BUTTKE TM, MCCUBREY JA AND OWEN TC. (1993). Use of an aqueous soluble tetrazolium/formazan assay to measure viability and proliferation of lymphokine-dependent cell lines. *J. Immunol. Methods*, **157**, 233–240.

COMBRIATO G AND KLOBECK H-G. (1991). V$\lambda$ and J$\lambda$–C$\lambda$ gene segments of the human immunoglobulin $\lambda$ light chain locus are separated by 14 kb and rearrange by a deletion mechanism. *Eur. J. Immunol.*, **21**, 1513–1522.

DORREEN MS, HABESHAW JA, WRIGLEY PF AND LISTER TA. (1982). Distribution of T-lymphocyte subsets in Hodgkin's disease characterized by monoclonal antibodies. *Br. J. Cancer*, **45**, 491–497.

DUCHOSAL MA, EMING SA, FISHER P, LETURCQ D, BARBAS III CF, MCCONAHEY PJ, CAOTHIEN RH, THORNTON GB, DIXON FJ AND BURTON DR. (1992). Immunization of hu-PBL-SCID mice and the rescue of human monoclonal Fab fragments through combinatorial libraries. *Nature*, **355**, 258–262.

ERB K, BORUP-CHRISTENSEN P, DITZEL H, CHEMNITZ J, HAAS H AND JENSENIUS JC. (1992). Characterization of a human-human hybridoma antibody, C-OU1, directed against a colon tumor-associated antigen. *Hybridoma*, **11**, 121–134.

FOUNG S, PERKINS S, KAFADAR K, GESSNER P AND ZIMMERMANN U. (1990). Development of microfusion techniques to generate human hybridomas. *J. Immunol. Methods*, **134**, 35–42.

GREEN LL, HARDY MC, MAYNARD-CURRIE CE, TSUDA H, LOUIE DM, MENDEZ MJ, ABDERRAHIM H, NOGUCHI M, SMITH DH, ZENG Y, DAVID NE, SASAI H, GARZA D, BRENNER DG, HALES JF, MCGUINNESS RP, CAPON DJ, KLAPHOLZ S AND JAKOBOVITS A. (1994). Antigen-specific human monoclonal antibodies from mice engineering with human Ig heavy and light chain YACs. *Nat. Genet.*, **7**, 13–21.

HANNA MG JR, HASPEL MV, MCCABE RP, MURRAY JH AND POMATO N. (1991). Development and application of human monoclonal antibodies. *Antibody, Immunoconjugates, Radiopharm.*, **4**, 67–75.

HASPEL MV, MCCABE RP, POMATO N, JANESCH NJ, KNOWLTON JV, PETERS LC, HOOVER HC JR AND HANNA MG JR. (1985). Generation of tumor cell-reactive human monoclonal antibodies using peripheral blood lymphocytes from actively immunized colorectal carcinoma patients. *Cancer Res.*, **45**, 3951–3961.

HIRANO H AND WATANABE T. (1990). Microsequencing of proteins electrotransferred onto immobilizing matrices from polyacrylamide gel electrophoresis: application to an insoluble protein. *Electrophoresis*, **11**, 573–580.

IIZASA T, YAMAGUCHI Y, TAGAWA M, SAITO H, FUJISAWA T, KATO K AND TANIGUCHI M. (1990). Establishment of human monoclonal antibody recognizing a new tumor-associated antigen from a patient with small cell lung carcinoma. *Hybridoma*, **9**, 211–219.

JAMES K AND BELL GT. (1987). Human monoclonal antibody production: current status and future prospects. *J. Immunol. Methods*, **100**, 5–40.

KÖHLER G AND MILSTEIN C. (1975). Continuous cultures of fused cell secreting antibody of predetermined specificity. *Nature*, **256**, 495–497.

KRISTENSEN P, JOHNSEN AH, UERKVITZ W, TANAKA K AND HENDIL KB. (1994). Human proteasome subunits from 2-dimensional gels identified by partial sequencing. *Biochem. Biophys. Res. Commun.*, **205**, 1785–1789.

LAEMMLI UK. (1970). Cleavage of structural proteins during the assembly of the head of the bacteriophage $T_4$. *Nature*, **227**, 680–685.

LONBERG N, TAYLOR LD, HARDING FA, TROUNSTINE M, HIGGINS KM, SCHRAMM SR, KUO C-C, MASHAYEKH R, WYMORE K, MCCABE JG, MUNOZ-O'REGAN D, O'DONELL SL, LAPACHET ESG, BEGOECHEA T, FISHWILD DM, CARMACK CE, KAY RM AND HUSZAR D. (1994). Antigen-specific human antibodies from mice comprising four distinct genetic modifications. *Nature*, **368**, 856–859.

MCCAFFERTY J, GRIFFITHS AD, WINTER G AND CHISWELL DJ. (1990). Phage antibodies: filamentous phage displaying antibody variable domains. *Nature*, **348**, 552–554.

MCCUNE JM, NAMIKAWA R, KANESHIMA H, SHULTZ JD, LIEBERMAN M AND WEISSMAN IL. (1988). The SCID-hu mouse: murine model for the analysis of human hematolymphoid differentiation and function. *Science*, **241**, 1632–1639.

MOLL R, FRANKE WW, SCHILLER DL, GEIGER B AND KREPLER R. (1982). The catalog of human cytokeratins: patterns of expression in normal epithelia, tumors and cultured cells. *Cell*, **31**, 11–24.

MURAKAMI H, HASHIZUME S, OHASHI H, SHINOHARA K, YASUMOTO K, NOMOTO K AND OMURA H. (1985). Human–human hybridomas secreting antibodies specific to human lung carcinoma. *In vitro Cell. Devel. Biol.*, **21**, 593–596.

PETERS J-M. (1994). Proteasomes: protein degradation machines of the cell. *Trends Biol. Sci.*, **19**, 377–382.

PETERS J-M, CEJKA Z, HARRIS JR, KLEINSCHMIDT JA AND BAUMEISTER W. (1993). Structural features of the 26S proteasome complex. *J. Mol. Biol.*, **234**, 932–937.

POMATO N, MURRAY JH, BOS E, HASPEL MV, MCCABE RP AND HANNA MG JR. (1989). Identification and characterization of a human colon tumor-associated antigen, CTAA 16-88, recognized by a human monoclonal antibody. In *Human Tumor Antigens and Specific Tumor Therapy*, Metzgar RS and Mitchell MS (eds), pp. 127–136. Alan R Liss: New York.

RAMAEKERS F, HUYSMANS A, MOESKER O, KANT A, JAP P, HERMAN C AND VOOIJS P. (1983). Monoclonal antibody to keratin filaments, specific for glandular epithelia and their tumors: use in surgical pathology. *Lab. Invest.*, **49**, 353–361.

RECHSTEINER M, HOFFMAN L AND DUBIEL W. (1993). The multicatalytic and 26S proteases. *J. Biol. Chem.*, **268**, 6065–6068.

RIVETT AJ. (1993). Proteasomes: multicatalytic proteinase complexes. *Biochem. J.*, **291**, 1–10.

RUGHETTI A, TURCHI V, GHETTI CA, SCAMBIA G, PANICI PB, RONCUCCI G, MANCUSO S, FRATI L AND NUTI M. (1993). Human B-cell immune response to the polymorphic epithelial mucin. *Cancer Res.*, **53**, 2457–2459.

RUUSKANEN O, PITTARD III WB, MILLER K, PIERCE G, SORENSEN RU AND POLMAR SH. (1980). *Staphylococcus aureus* Cowan I-induced immunoglobulin production in human cord blood lymphocytes. *J. Immunol.*, **125**, 411–413.

SATO Y, MUKAI K, WATANABE S, GOTO M AND SHIMOSATO Y. (1986). The AMeX method. A simplified technique of tissue processing and paraffin embedding with improved preservation of antigens for immunostaining. *Am. J. Pathol.*, **125**, 431–435.

SCHUURMAN RKB, GELFAND EW AND DOSCH H-M. (1980). Polyclonal activation of human lymphocytes *in vitro*. *J. Immunol.*, **125**, 820–826.

STEVENS MG AND OLSEN SC. (1993). Comparative analysis of using MTT and XTT in colorimetric assays for quantitating bovine neutrophil bactericidal activity. *J. Immunol. Methods*, **157**, 225–231.

TOWBIN H, STAEHELIN T AND GORDON J. (1979). Electrophoretic transfer of proteins from polyacrylamide gels to nitrocellulose sheets: procedure and some applications. *Proc. Natl Acad. Sci. USA*, **76**, 4350–4354.

TYTGAT KMAJ, BÜLLER HA, OPDAM FJM, KIM YS, EINERHAND AWC AND DEKKER J. (1994). Biosynthesis of human colonic mucin: Muc2 is the prominent secretory mucin. *Gastroenterology*, **107**, 1352–1363.

UCHIYAMA K, SAITO H, TOKUHISA T, IMAI K AND TANIGUCHI M. (1987). High frequency of loss of human kappa light chain expression in mouse–human heterohybridomas. *Hybridoma*, **6**, 645–654.

UMETSU DT AND GEHA RS. (1987). *In vitro* production of antibody in cultures of human peripheral blood lymphocytes. *Methods Enzymol.*, **150**, 309–316.

YOSHINARI K, ARAI K, KIMURA H, MATSUMOTO K AND YAMAGUCHI Y. (1996). Efficient production of IgG human monoclonal antibodies by lymphocytes stimulated by lipopolysaccharide, pokeweed mitogen, and interleukin 4. *In Vitro Cell. Devel. Biol.*, **32** (in press).

ZEBEDEE SL, BARBAS III CF, HOM Y-L, CAOTHIEN RH, GRAFF R, DEGRAW J, PYATI J, LAPOLLA R, BURTON DR, LERNER RA AND THORNTON GB. (1992). Human combinatorial antibody libraries to hepatitis B surface antigen. *Proc. Natl Acad. Sci. USA*, **89**, 3175–3179.

ZIEGLER-HEITBROCK HWL AND ULEVITCH RJ. (1993). CD14: cell surface receptor and differentiation marker. *Immunol. Today*, **14**, 121–125.

ZIMMERMANN U, GESSNER P, SCHNETTLER R, PERKINS S AND FOUNG S. (1990). Efficient hybridization of mouse–human cell lines by means of hypo-osmolar electrofusion. *J. Immunol. Methods*, **134**, 43–50.


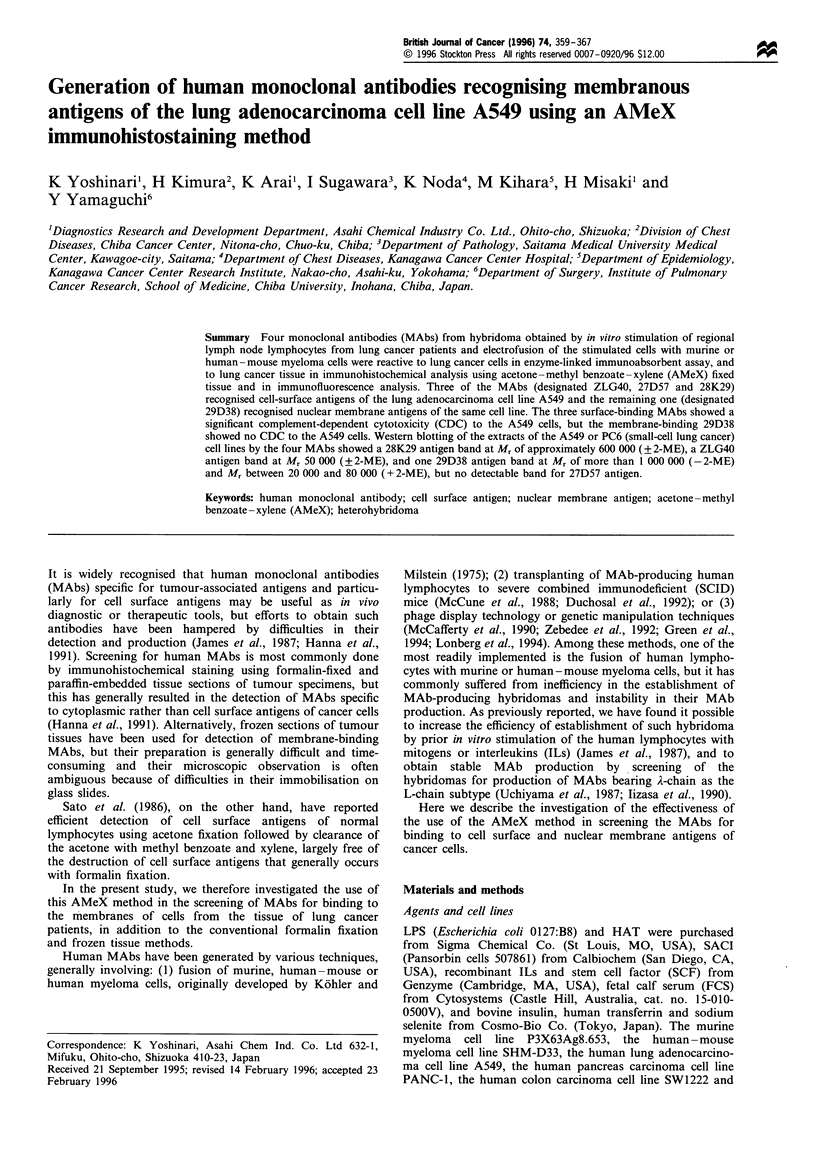

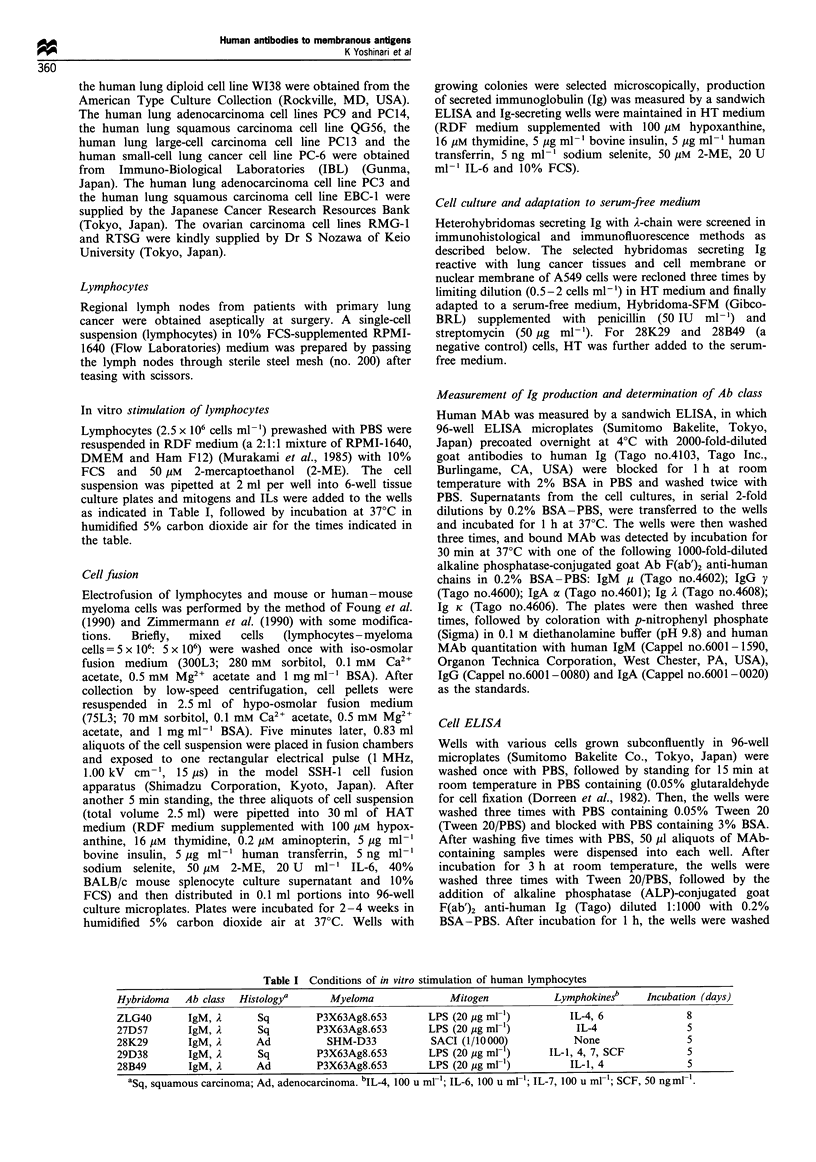

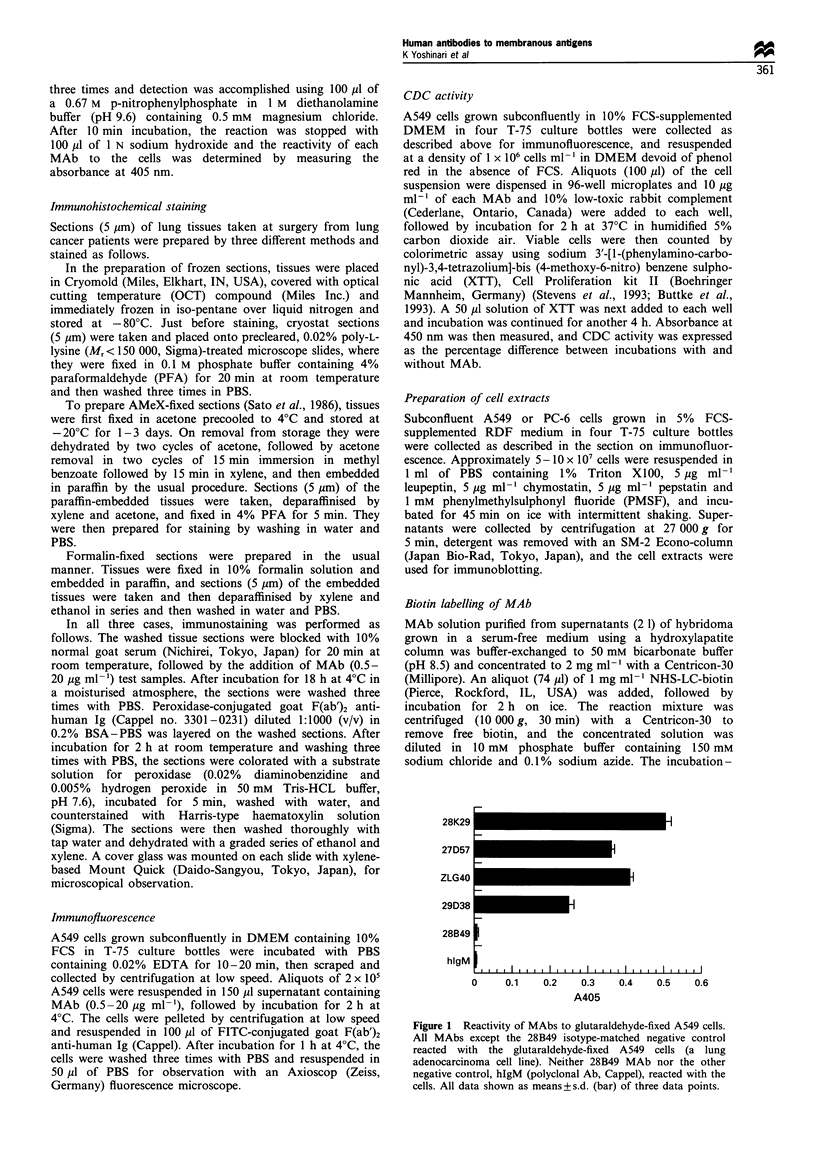

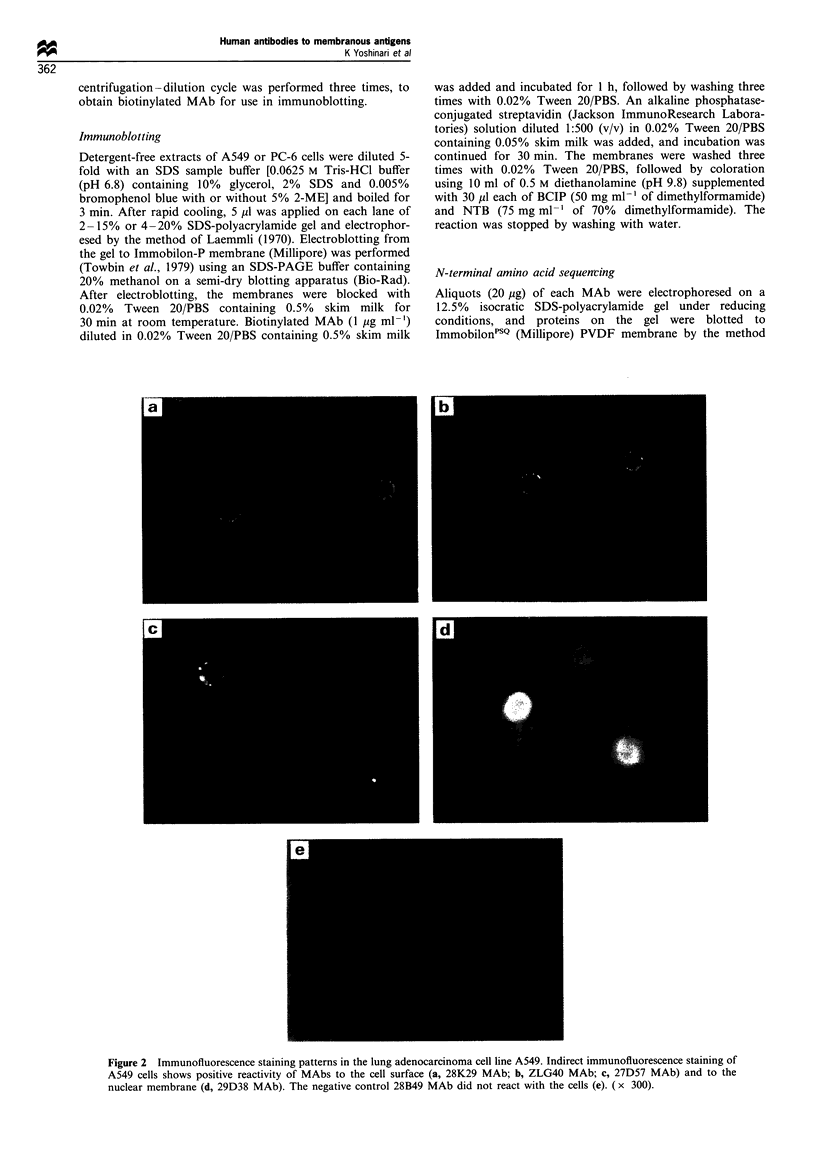

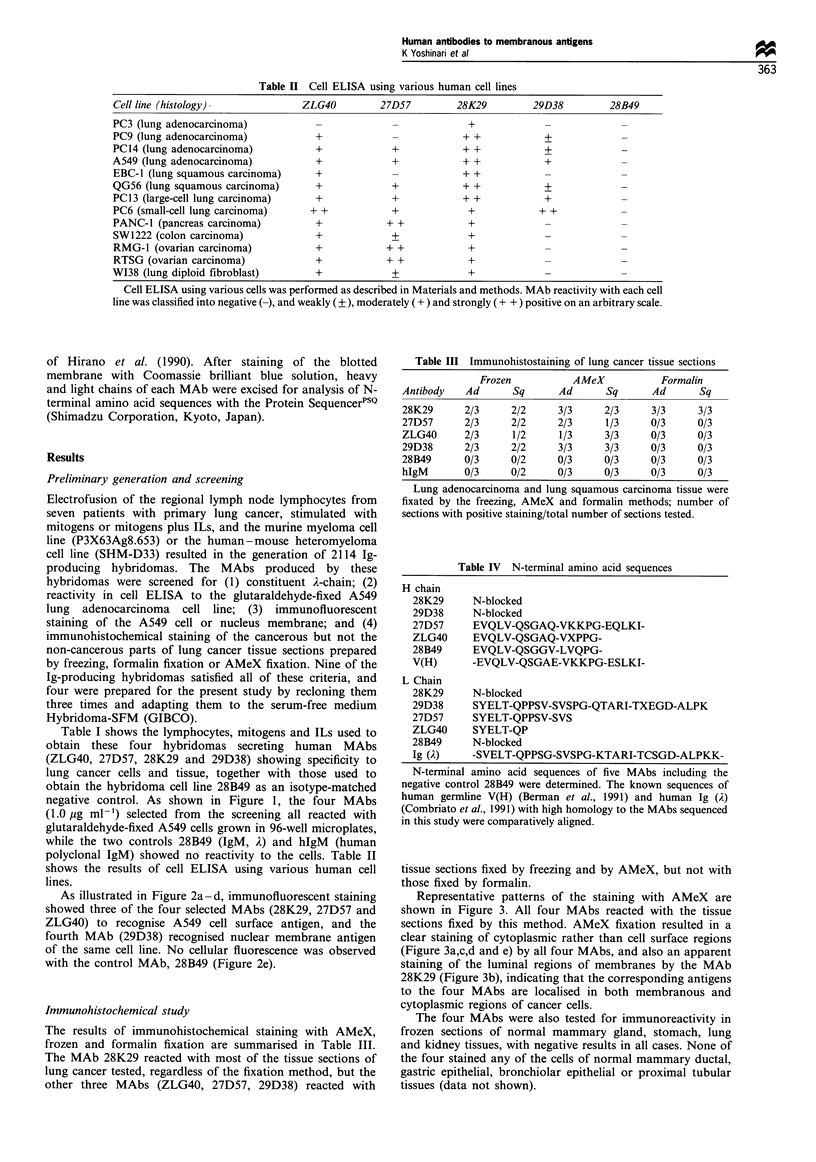

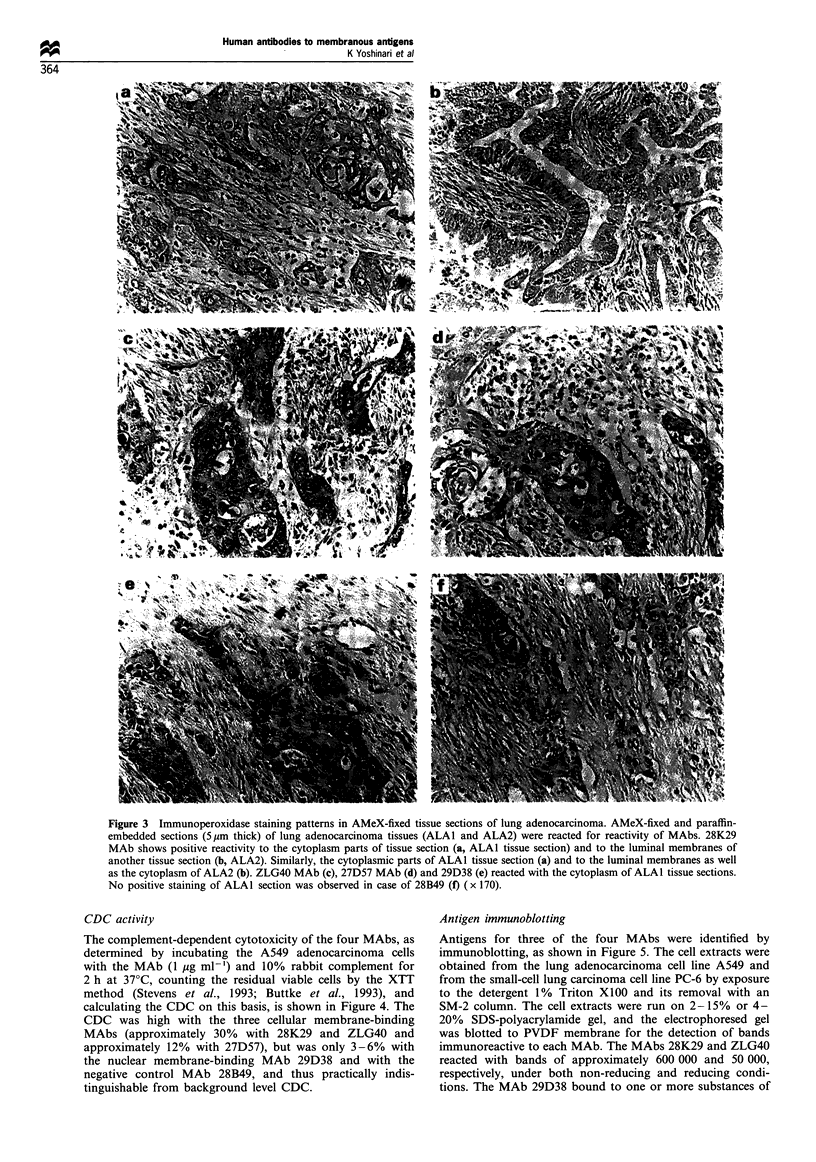

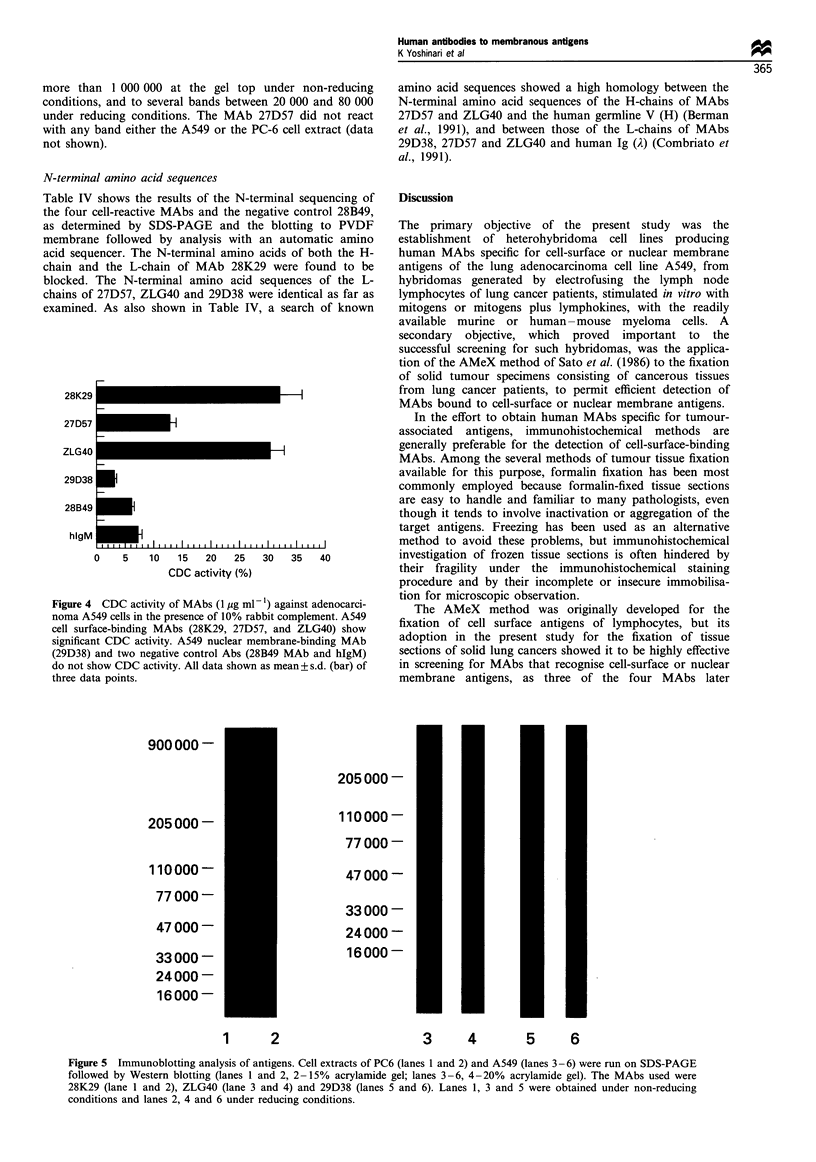

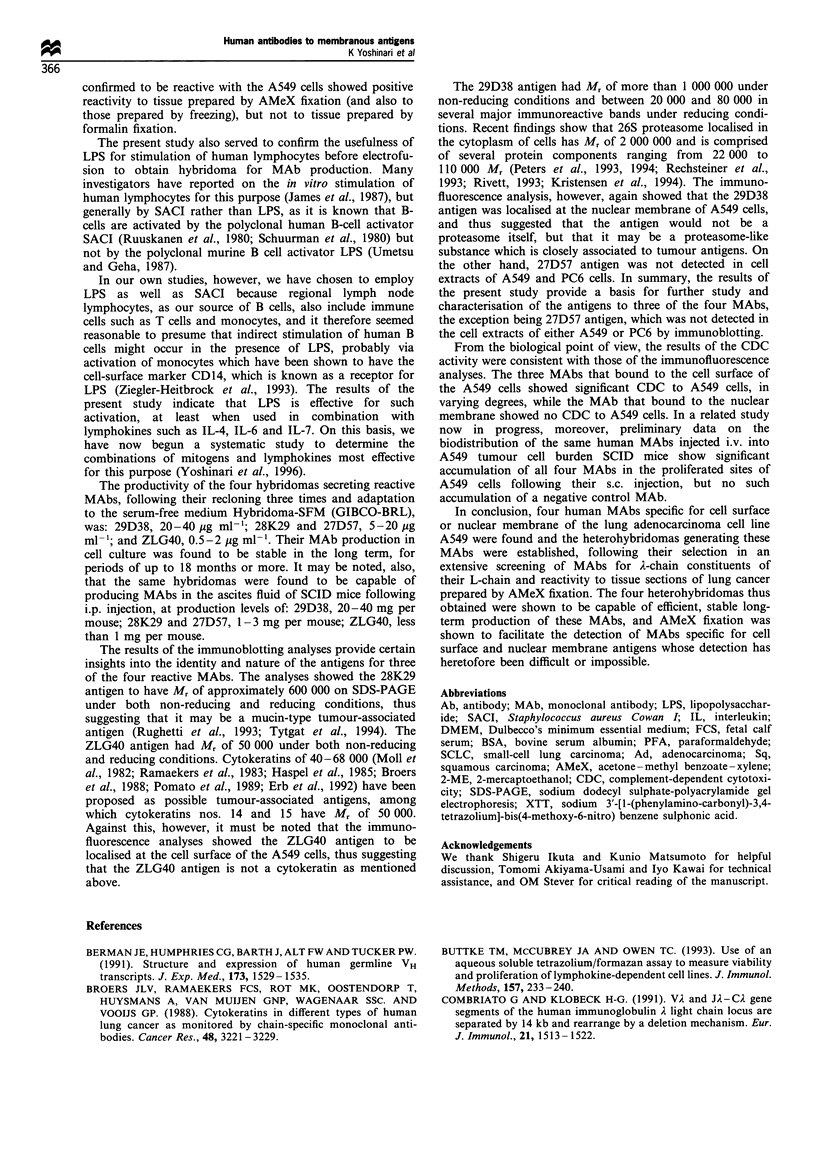

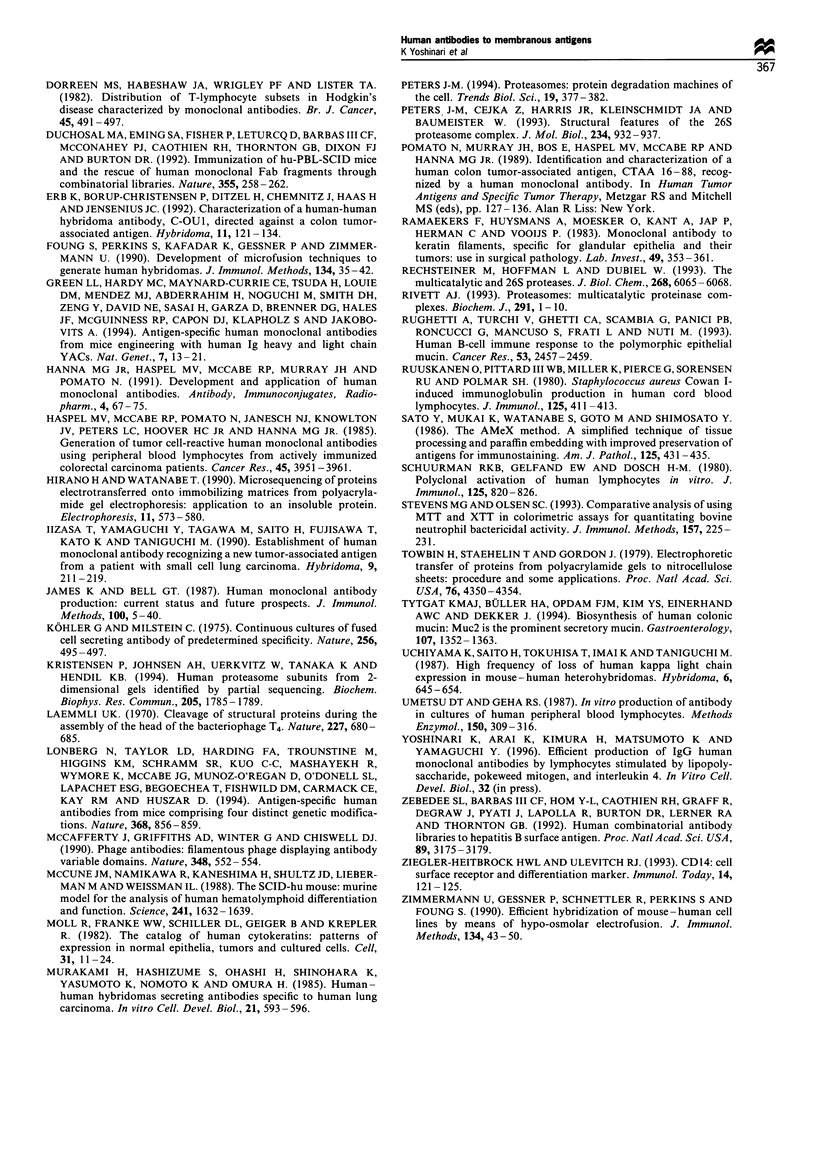


## References

[OCR_00923] Berman J. E., Humphries C. G., Barth J., Alt F. W., Tucker P. W. (1991). Structure and expression of human germline VH transcripts.. J Exp Med.

[OCR_00929] Broers J. L., Ramaekers F. C., Rot M. K., Oostendorp T., Huysmans A., van Muijen G. N., Wagenaar S. S., Vooijs G. P. (1988). Cytokeratins in different types of human lung cancer as monitored by chain-specific monoclonal antibodies.. Cancer Res.

[OCR_00933] Buttke T. M., McCubrey J. A., Owen T. C. (1993). Use of an aqueous soluble tetrazolium/formazan assay to measure viability and proliferation of lymphokine-dependent cell lines.. J Immunol Methods.

[OCR_00945] Combriato G., Klobeck H. G. (1991). V lambda and J lambda-C lambda gene segments of the human immunoglobulin lambda light chain locus are separated by 14 kb and rearrange by a deletion mechanism.. Eur J Immunol.

[OCR_00953] Dorreen M. S., Habeshaw J. A., Wrigley P. F., Lister T. A. (1982). Distribution of T-lymphocyte subsets in Hodgkin's disease characterized by monoclonal antibodies.. Br J Cancer.

[OCR_00960] Duchosal M. A., Eming S. A., Fischer P., Leturcq D., Barbas C. F., McConahey P. J., Caothien R. H., Thornton G. B., Dixon F. J., Burton D. R. (1992). Immunization of hu-PBL-SCID mice and the rescue of human monoclonal Fab fragments through combinatorial libraries.. Nature.

[OCR_00967] Erb K., Borup-Christensen P., Ditzel H., Chemnitz J., Haas H., Jensenius J. C. (1992). Characterization of a human-human hybridoma antibody, C-OU1, directed against a colon tumor-associated antigen.. Hybridoma.

[OCR_00972] Foung S., Perkins S., Kafadar K., Gessner P., Zimmermann U. (1990). Development of microfusion techniques to generate human hybridomas.. J Immunol Methods.

[OCR_00977] Green L. L., Hardy M. C., Maynard-Currie C. E., Tsuda H., Louie D. M., Mendez M. J., Abderrahim H., Noguchi M., Smith D. H., Zeng Y. (1994). Antigen-specific human monoclonal antibodies from mice engineered with human Ig heavy and light chain YACs.. Nat Genet.

[OCR_00992] Haspel M. V., McCabe R. P., Pomato N., Janesch N. J., Knowlton J. V., Peters L. C., Hoover H. C., Hanna M. G. (1985). Generation of tumor cell-reactive human monoclonal antibodies using peripheral blood lymphocytes from actively immunized colorectal carcinoma patients.. Cancer Res.

[OCR_00998] Hirano H., Watanabe T. (1990). Microsequencing of proteins electrotransferred onto immobilizing matrices from polyacrylamide gel electrophoresis: application to an insoluble protein.. Electrophoresis.

[OCR_01002] Iizasa T., Yamaguchi Y., Tagawa M., Saito H., Fujisawa T., Kato K., Taniguchi M. (1990). Establishment of human monoclonal antibody recognizing a new tumor-associated antigen from a patient with small cell lung carcinoma.. Hybridoma.

[OCR_01009] James K., Bell G. T. (1987). Human monoclonal antibody production. Current status and future prospects.. J Immunol Methods.

[OCR_01022] Kristensen P., Johnsen A. H., Uerkvitz W., Tanaka K., Hendil K. B. (1994). Human proteasome subunits from 2-dimensional gels identified by partial sequencing.. Biochem Biophys Res Commun.

[OCR_01014] Köhler G., Milstein C. (1975). Continuous cultures of fused cells secreting antibody of predefined specificity.. Nature.

[OCR_01025] Laemmli U. K. (1970). Cleavage of structural proteins during the assembly of the head of bacteriophage T4.. Nature.

[OCR_01030] Lonberg N., Taylor L. D., Harding F. A., Trounstine M., Higgins K. M., Schramm S. R., Kuo C. C., Mashayekh R., Wymore K., McCabe J. G. (1994). Antigen-specific human antibodies from mice comprising four distinct genetic modifications.. Nature.

[OCR_01041] McCafferty J., Griffiths A. D., Winter G., Chiswell D. J. (1990). Phage antibodies: filamentous phage displaying antibody variable domains.. Nature.

[OCR_01046] McCune J. M., Namikawa R., Kaneshima H., Shultz L. D., Lieberman M., Weissman I. L. (1988). The SCID-hu mouse: murine model for the analysis of human hematolymphoid differentiation and function.. Science.

[OCR_01050] Moll R., Franke W. W., Schiller D. L., Geiger B., Krepler R. (1982). The catalog of human cytokeratins: patterns of expression in normal epithelia, tumors and cultured cells.. Cell.

[OCR_01059] Murakami H., Hashizume S., Ohashi H., Shinohara K., Yasumoto K., Nomoto K., Omura H. (1985). Human-human hybridomas secreting antibodies specific to human lung carcinoma.. In Vitro Cell Dev Biol.

[OCR_01069] Peters J. M., Cejka Z., Harris J. R., Kleinschmidt J. A., Baumeister W. (1993). Structural features of the 26 S proteasome complex.. J Mol Biol.

[OCR_01064] Peters J. M. (1994). Proteasomes: protein degradation machines of the cell.. Trends Biochem Sci.

[OCR_01082] Ramaekers F., Huysmans A., Moesker O., Kant A., Jap P., Herman C., Vooijs P. (1983). Monoclonal antibody to keratin filaments, specific for glandular epithelia and their tumors. Use in surgical pathology.. Lab Invest.

[OCR_01087] Rechsteiner M., Hoffman L., Dubiel W. (1993). The multicatalytic and 26 S proteases.. J Biol Chem.

[OCR_01088] Rivett A. J. (1993). Proteasomes: multicatalytic proteinase complexes.. Biochem J.

[OCR_01094] Rughetti A., Turchi V., Ghetti C. A., Scambia G., Panici P. B., Roncucci G., Mancuso S., Frati L., Nuti M. (1993). Human B-cell immune response to the polymorphic epithelial mucin.. Cancer Res.

[OCR_01101] Ruuskanen O., Pittard W. B., Miller K., Pierce G., Sorensen R. U., Polmar S. H. (1980). Staphylococcus aureus Cowan I-induced immunoglobulin production in human cord blood lymphocytes.. J Immunol.

[OCR_01106] Sato Y., Mukai K., Watanabe S., Goto M., Shimosato Y. (1986). The AMeX method. A simplified technique of tissue processing and paraffin embedding with improved preservation of antigens for immunostaining.. Am J Pathol.

[OCR_01112] Schuurman R. K., Gelfand E. W., Dosch H. M. (1980). Polyclonal activation of human lymphocytes in vitro. I. Characterization of the lymphocyte response to a T cell-independent B cell mitogen.. J Immunol.

[OCR_01117] Stevens M. G., Olsen S. C. (1993). Comparative analysis of using MTT and XTT in colorimetric assays for quantitating bovine neutrophil bactericidal activity.. J Immunol Methods.

[OCR_01123] Towbin H., Staehelin T., Gordon J. (1979). Electrophoretic transfer of proteins from polyacrylamide gels to nitrocellulose sheets: procedure and some applications.. Proc Natl Acad Sci U S A.

[OCR_01130] Tytgat K. M., Büller H. A., Opdam F. J., Kim Y. S., Einerhand A. W., Dekker J. (1994). Biosynthesis of human colonic mucin: Muc2 is the prominent secretory mucin.. Gastroenterology.

[OCR_01135] Uchiyama K., Saito H., Tokuhisa T., Imai K., Taniguchi M. (1987). High frequency of loss of human kappa light chain expression in mouse-human heterohybridomas.. Hybridoma.

[OCR_01141] Umetsu D. T., Geha R. S. (1987). In vitro production of antibody in cultures of human peripheral blood lymphocytes.. Methods Enzymol.

[OCR_01153] Zebedee S. L., Barbas C. F., Hom Y. L., Caothien R. H., Graff R., DeGraw J., Pyati J., LaPolla R., Burton D. R., Lerner R. A. (1992). Human combinatorial antibody libraries to hepatitis B surface antigen.. Proc Natl Acad Sci U S A.

[OCR_01158] Ziegler-Heitbrock H. W., Ulevitch R. J. (1993). CD14: cell surface receptor and differentiation marker.. Immunol Today.

[OCR_01163] Zimmermann U., Gessner P., Schnettler R., Perkins S., Foung S. K. (1990). Efficient hybridization of mouse-human cell lines by means of hypo-osmolar electrofusion.. J Immunol Methods.

